# Suppression of plant defense responses by extracellular metabolites from *Pseudomonas syringae* pv. *tabaci* in *Nicotiana benthamiana*

**DOI:** 10.1186/1471-2229-13-65

**Published:** 2013-04-18

**Authors:** Seonghee Lee, Dong Sik Yang, Srinivasa Rao Uppalapati, Lloyd W Sumner, Kirankumar S Mysore

**Affiliations:** 1 The Samuel Roberts Noble Foundation, Plant Biology Division, Ardmore, OK, 73401, USA

**Keywords:** *Nicotiana benthamiana*, *Pseudomonas syringae* pv. *tabaci*, Extracellular metabolites, Hypersensitive response (HR), Stomata, Nonhost resistance

## Abstract

**Background:**

*Pseudomonas syringae* pv. *tabaci* (*Pstab*) is the causal agent of wildfire disease in tobacco plants. Several pathovars of *Pseudomonas syringae* produce a phytotoxic extracellular metabolite called coronatine (COR). COR has been shown to suppress plant defense responses. Interestingly, *Pstab* does not produce COR but still actively suppresses early plant defense responses. It is not clear if *Pstab* produces any extracellular metabolites that actively suppress early defense during bacterial pathogenesis.

**Results:**

We found that the *Pstab* extracellular metabolite extracts (*Pstab* extracts) remarkably suppressed stomatal closure and nonhost hypersensitive response (HR) cell death induced by a nonhost pathogen, *P. syringae* pv. *tomato* T1 (*Pst* T1), in *Nicotiana benthamiana*. We also found that the accumulation of nonhost pathogens, *Pst* T1 and *P. syringae* pv*. glycinea* (*Psgly*), was increased in *N. benthamiana* plants upon treatment with *Pstab* extracts . The HR cell death induced by Pathogen-Associated Molecular Pattern (INF1), gene-for-gene interaction (*Pto*/*AvrPto* and *Cf-9*/*AvrCf-9*) and ethanol was not delayed or suppressed by *Pstab* extracts. We performed metabolite profiling to investigate the extracellular metabolites from *Pstab* using UPLC-qTOF-MS and identified 49 extracellular metabolites from the *Pstab* supernatant culture. The results from gene expression profiling of *PR-1, PR-2*, *PR-5*, *PDF1.2*, *ABA1*, *COI1*, and *HSR203J* suggest that *Pstab* extracellular metabolites may interfere with SA-mediated defense pathways.

**Conclusions:**

In this study, we found that *Pstab* extracts suppress plant defense responses such as stomatal closure and nonhost HR cell death induced by the nonhost bacterial pathogen *Pst* T1 in *N. benthamiana*.

## Background

Foliar bacterial phytopathogens such as the *Pseudomonas syringae* species survive on the plant leaf surface as epiphytes [1]. During the initial infection process, the bacterial pathogens produce virulence factors including effector proteins and secondary metabolites, to inactivate early plant defense responses such as stomata-based immunity [2,3] and hypersensitive response (HR) cell death at the site of infection [4]. The failure of early pathogen recognition delays initiation of the downstream defense cascade and results in the development of disease symptoms in plants. Therefore, the suppression of early plant defense responses is one of the important steps for bacterial pathogens to successfully colonize plant tissues, leading to disease.

It has long been thought that stomata are the passive portal for entry of phytopathogens. However, recent studies demonstrated that stomata play an active role in restricting bacterial invasion as part of the plant innate immune system [2,5]. Perception of multiple bacterial pathogen-associated molecular patterns (PAMPs), including flagellin, lipopolysaccharide (LPS) and elongation factor Tu (EF-Tu) induces closure of stomata in leaf epidermal peels of *Arabidopsis*[6]. It is now believed that stomatal closure is a common plant defense response initiated by the perception of bacterial PAMPs and limits bacterial invasion in plants. However, certain bacterial pathogens have evolved to deliver specific virulence factors such as coronatine (COR) to overcome PAMP-triggered immunity (PTI) and stomata-based defense.

COR is a nonhost-specific, non-proteinaceous virulence effector produced by several pathovars of *P. syringae*[7,8]. This is one of the most extensively studied phytobacterial secondary metabolites that modulate plant hormonal defense signaling and functions as a stomatal-based immunity suppressor. COR has structural and functional similarity to jasmonates including 12-oxo-phytodienoic acid (12-OPDA) and jasmonic acid-isoleucine (JA-Ile), and activates the JA pathway in Arabidopsis and tomato [9-11]. The virulent pathogen *P*. *syringae* pv. *tomato* strain DC3000 (*Pst* DC3000) produces COR on the plant surface to reopen closed stomata, allowing increased bacterial entry [2,3]. A *Pst* DC3000 mutant (*Pst* DC3118) that is deficient in COR production has severely attenuated virulence when dip- or spray-inoculated onto Arabidopsis and tomato leaves [3]. However, this defect can be restored in Arabidopsis mutants (*fls2*, *ost1* and *gpa1*) that are defective in abscisic acid (ABA)- and PAMP-regulated stomatal closure [12,13].

In contrast to the number of studies done for COR-producing bacterial pathogens, it has been largely overlooked that other pathovars of *P. syringae* without COR may also produce non-proteinaceous virulence factors to suppress plant innate immunity. *Xanthomonas campestris* pv. *campestris* (*Xcc*) that has a broad host range including *Brassicaceae* family is shown to overcome stomatal defense in Arabidopsis [14]. The extracellular metabolite secreted from *Xcc* is regulated by *rpf* (regulation of pathogenicity factor) gene cluster. The *rpf* mutant strains of *Xcc* were unable to reopen stomata, but the stomata closure was reverted when ethyl acetate extracts from *Xcc* culture supernatants were added to the mutant strains in Arabidopsis [14]. Two other *P. syringae* strains, *P. s*. pv. *tabaci* (*Pstab*) and *P. s*. pv. *tomato* strain T1 (*Pst* T1), do not produce COR, but these bacterial strains can actively reopen stomata in tobacco and tomato plants, respectively [3,5].

Hypersensitive response (HR) is another important form of early defense response against bacterial pathogens. HR is associated with defenses that are highly manifested by development of rapid cell death. A number of HR elicitors from bacterial pathogens have been described. Phytobacterial avirulent proteins (Avr) cause HR during incompatible interactions in plants containing corresponding plant resistance (*R*) genes (gene-for-gene resistance-mediated HR). Protein products of the *hrp* (hypersensitive response and pathogenicity) gene family cause HR in nonhost plants (nonhost disease resistance-mediated HR; nonhost HR). The nonhost HR cell death is the common phenomenon observed in many plants in response to non-adapted bacterial pathogens [15]. The bacterial effector proteins of *P. syringae* are injected into plant cells by the pathogen type III secretion system (TTSS) to suppress basal resistance in host plants [16,17]. The TTSS- and *Hrp*-deficient mutants cannot elicit nonhost HR cell death or be pathogenic on host plants [18]. Moreover, several effectors from *Pst* DC3000 play an important role in suppression of the *R*-gene mediated HR in tomato [19-21]. In addition, it has been also shown that the *Pst* DC3000 effector, AvrPto, suppresses nonhost HR cell death in *Nicotiana benthamiana* and tomato [22]. We recently showed that COR can also suppress HR induced by a nonhost pathogen in *N. benthmaina*[23].

In the current study, we report that the extracellular metabolite(s) from *Pstab* suppresses plant defense responses such as stomata-based immunity and hypersensitive response (HR) cell death. We performed extracellular metabolite profiling of *Pstab* by ultra high performance liquid chromatography coupled to hybrid quadrupole time-of-flight mass spectrometry (UHPLC-qTOF-MS) and isolated putative metabolites involved in the suppression of early plant defense responses in *N. benthamiana*. The patterns of plant defense gene expression suggest that the SA-mediated defense pathway may be modulated by extracellular metabolites from *Pstab*.

## Results

### Bacterial pathogens, *Pstab* and *Pst* T1, suppress early defense responses in their host plants, *N. benthamiana* and tomato, respectively

**Figure 1:**
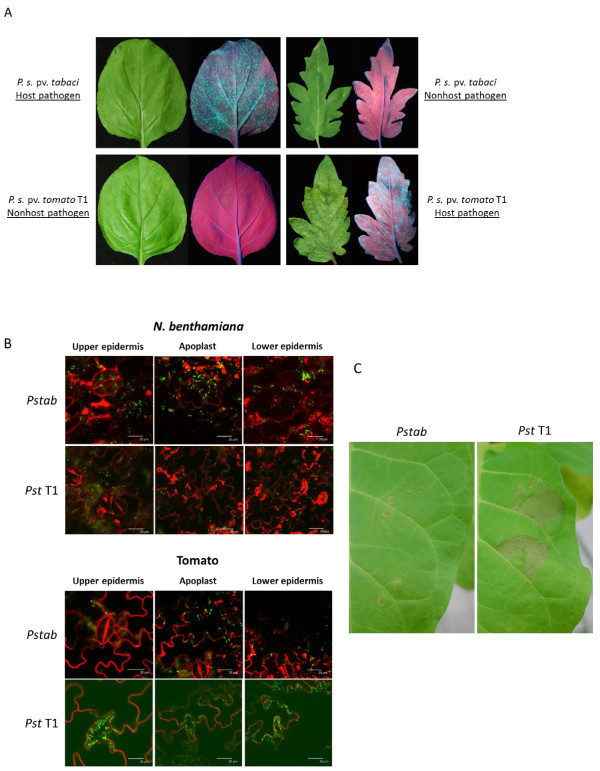
**Suppression of stomatal closure and hypersensitive response (HR) cell death by the host bacterial pathogen *Pstab *in *N. benthamiana*.** (**A**) Inoculation of GFP*uv*-expressing bacterial pathogen of *Pstab* (6×10^3^ CFU/ml) in *N. benthamiana* and *Pst* T1 (6×10^3^ CFU/ml) in tomato. Plants were spray inoculated with appropriate bacteria and photographed three days after inoculation. (**B**) Confocal microscopic images of GFP*uv*-expressing *Pstab* and *Pst* T1 on epidermal peels of *N. benthamiana* and tomato, respectively. *Pstab* in *N. benthamiana* and *Pst* T1 in tomato were able to reopen stomata 3 hrs after bacterial inoculation, localized around open stomata and in apoplastic space. Epidermal peels were stained with FM4-64 (Invitrogen, Grand Island, N.Y.) to visualize the plasma membrane. Several confocal images were taken from z-series by focusing the stomatal and apoplastic region. (**C**) Suppression of nonhost HR cell death by *Pstab* in *N. benthamiana*. *N. benthamiana* leaves were syringe-infiltrated with host or nonhost pathogens, *Pstab* or *Pst* T1, with a concentration of 2×10^6^ CFU/ml, respectively. Image was taken 16 hours after inoculation.

The early infection processes of *Pstab* in *N. benthamiana* and *Pst* T1 in tomato were determined using *GFPuv*-expressing bacteria [24]. Five days after spray inoculation, a number of fluorescent spots (bacterial colonization) at infection sites were observed under long-wavelength UV light in *N. benthamiana* with *Pstab* and in tomato with *Pst* T1 infection. In contrast, only a few fluorescent spots were detected on *N. benthamiana* leaves infected with *Pst* T1 and on tomato plants infected with *Pstab* (Figure 1A). We also observed that both *Pstab* and *Pst* T1 can reopen stomata and actively enter into apoplastic space in their respective host plants (Figure 1B). However, most stomata remained closed in tomato when inoculated with a nonhost pathogen, *Pstab.* Similarly, most *N. benthamiana* stomata remained closed when inoculated with a nonhost pathogen, *Pst* T1. In addition, the inoculation of *Pst* T1 induced typical nonhost HR cell death on *N. benthamiana* leaves within 20 hrs, while the host pathogen *Pstab* did not induce any visible cell death at that time point (Figure 1C). We speculate that the nonhost HR cell death in *N. benthamiana* is triggered either by PAMPs or effectors of the nonhost pathogen, *Pst* T1. Such PAMP- and/or effector-triggered HR is probably suppressed during *Pstab*-*N. benthamiana* interaction, thus resulting in disease symptoms after inoculation. These results suggested that *Pstab* produces host-specific virulence factor(s) to inactivate stomatal closure and probably suppress PAMP- and/or effector-triggered HR in *N. benthamiana*. This prompted us to investigate if any extracellular metabolite(s) from *Pstab* can inactivate some plant defense responses.

### Extracellular nonpolar metabolites from *Pstab* suppress stomatal closure induced by the nonhost bacterial pathogen *Pst* T1

**Figure 2:**
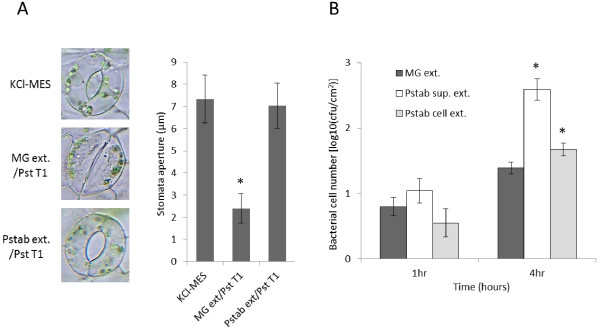
**Suppression of stomatal defense by the metabolite extract from *Pstab *supernatant in *N. benthamiana*.** (**A**) Suppression of stomatal closure by *Pstab* extracts in *N. benthamiana* epidermal peels. KCl-MES; stomata opening buffer, MG ext/*Pst* T1; bacterial suspension of *Pst* T1 with MG media extracts, and *Pstab* ext/*Pst* T1; bacterial suspension of *Pst* T1 with *Pstab* extracts. (**B**) Ability of *Pst* T1 migration through stomata and a number of bacterial cells of *Pst* T1 in apoplastic space. Detached leaves of *N. benthamiana* were floated on *Pst* T1 suspension (1×10^7^ CFU/ml) along with MG media, *Pstab* supernatant, and *Pstab* cell pellet extracts, and samples were collected at 1 hr and 4 hrs after incubation. Bars represent the means ± standard deviation (SD) (P<0.05, student’s *t-test*).

We prepared the *Pstab* extracts from bacterial culture to investigate whether the observed suppression of stomatal defense (shown in Figure 1B) is due to the metabolites secreted out of bacteria. The stomatal closure induced by the nonhost pathogen *Pst* T1 was suppressed when *Pst* T1 was mixed with the extracellular metabolites in the ethyl acetate extract of *Pstab* culture supernatant prior to inoculation in *N. benthamiana* (Figure 2A). To determine if the metabolites that suppress stomatal closure are extracellular or intracellular products, the *Pstab* extracts were individually prepared from both the culture supernatant and bacterial cell pellets. Detached *N. benthamiana* leaves were floated on a bacterial suspension of *Pst* T1 mixed with extracts from either the minimal growth medium (MG; control) or the *Pstab* supernatant (*Pstab* sup. ext.), or the *Pstab* bacterial cell pellet (*Pstab* cell ext.). The *Pst* T1 cell numbers in the leaf apoplast were significantly higher (due to more entry of bacteria through stomata) at 4 hrs after incubation of *Pst* T1 mixed with *Pstab* supernatant when compared to bacterial numbers in the leaf treated with *Pst* T1 mixed with *Pstab* cell extract or the control (Figure 2B). These findings suggest that *Pstab* supernatant extracts function to suppress stomatal closure. We also detected a slight increase in bacterial entry with cell extract compared to MG extracts, possibly due to the presence of remaining small amounts of *Pstab* extracellular extracts in bacterial cell pellet.

### *Pstab* extracts suppress hypersensitive response (HR) cell death triggered by nonhost pathogens

**Figure 3:**
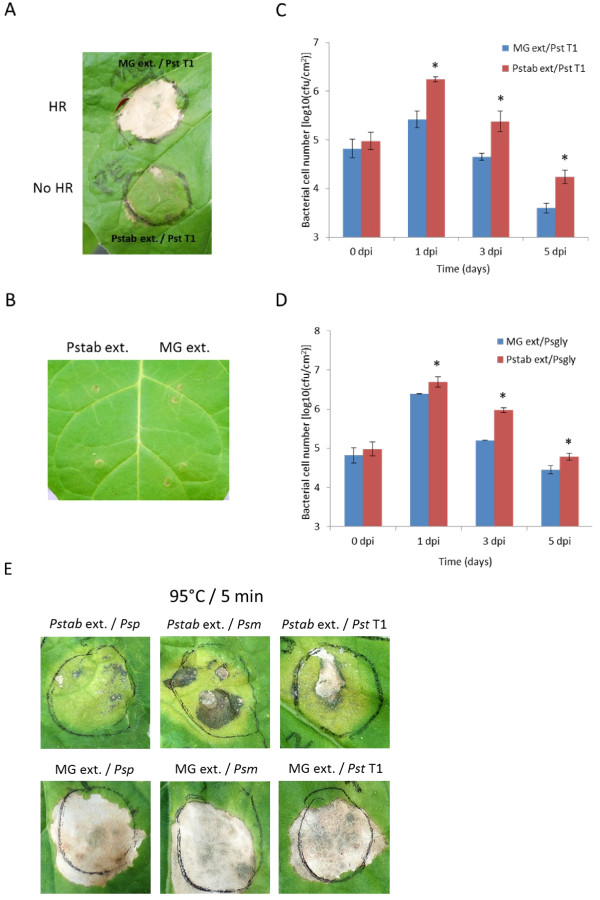
**Suppression of nonhost HR cell death and enhancement of bacterial multiplication by *Pstab *extracts in *N. benthamiana*.** (**A**) Suppression of nonhost HR cell death by the ethyl acetate extract from *Pstab* culture supernatant (*Pstab* ext.). *Pst* T1 (2.1 x 10^6^ CFU/ml) was infiltrated in *N. benthamiana* leaves, and photo was taken at 48 hpi. (**B**) Non-phytotoxin activity of *Pstab* extracts in *N. benthamiana* leaf. A high concentration of the *Pstab* extracts was used, 24 μl/ml instead of 6 μl/ml. Photographs were taken three days after infiltration. (**C** and **D**) Bacterial multiplications of nonhost pathogens *P. s.* pv. *tomato* T1 (*Pst* T1) and *P. s*. pv. *glycinea* (*Psgly*) after inoculation with *Pstab* extracts in *N. benthamiana*. *Pst* T1 (**C**) and *Psgly* (**D**) (3×10^4^ CFU/ml) were inoculated with *Pstab* extracts, and the bacterial growth was measured. The experiment was repeated twice (three replications for each experiment) with similar results. Bars represent the means ± standard deviation (SD) (P<0.05, student’s *t-test*). (**E**) Temperature stability of *Pstab* extracts and suppression of nonhost HR cell death for three nonhost pathogens, *P. s.* pv. *phaseolicola* (*Psp*), *P. s.* pv. *maculicola* (*Psm*), and *Pst* T1. *Pstab* extracts were boiled (5 min/95°C) and co-infiltrated with the bacterial suspensions. Photographs were taken at 24 hpi.

Nonhost disease resistance is the common plant defense mechanism which protects plants from various potential pathogens. HR is a common phenomenon for nonhost resistance observed in plant-bacterial pathogen interactions. We further tested whether the metabolites secreted from *Pstab* (*Pstab* extracts) can also overcome nonhost HR cell death induced by *Pst* T1 in *N. benthamiana*. HR occurred within 16 hrs of syringe infiltration of *Pst* T1 (2.1 × 10^6^ CFU/ml) in *N. benthamiana*. Interestingly, the nonhost HR cell death was suppressed when *Pst* T1 was infiltrated along with *Pstab* extracts. Furthermore, when *Pstab* extracts alone were infiltrated, there was no visible symptom on the *N. benthamiana* leaf (Figure 3A and 3B).

To determine if *Pstab* extracts play a role in virulence, we examined the growth of two nonhost bacterial pathogens, *Pst* T1 and *P. syringae* pv*. glycinea* (*Psgly*), in the *N. benthamiana* leaves in the presence of *Pstab* extracts. After co-inoculation of *Pst* T1 or *Psgly* with *Pstab* extracts, the bacterial growth was significantly higher on the inoculated area of both *Pst* T1 + *Pstab* extracts and *Psgly* + *Pstab* extracts than in the co-inoculated area of *Pst* T1 or *Psgly* with MG medium extracts (mock) (Figure 3C and 3D). Since *N. benthamiana* is a nonhost for both *Psgly* and *Pst* T1, their populations gradually decreased two days after inoculation in the mock control. The bacterial populations of *Psgly* and *Pst* T1 with *Pstab* extracts also decreased, but the populations were higher than the population after the mock inoculation. No visible disease symptoms were found in either *Psgly* + *Pstab* extracts or *Pst* T1 + *Pstab* extracts in the inoculated area of the leaves when compared to mock controls.

**Figure 4:**
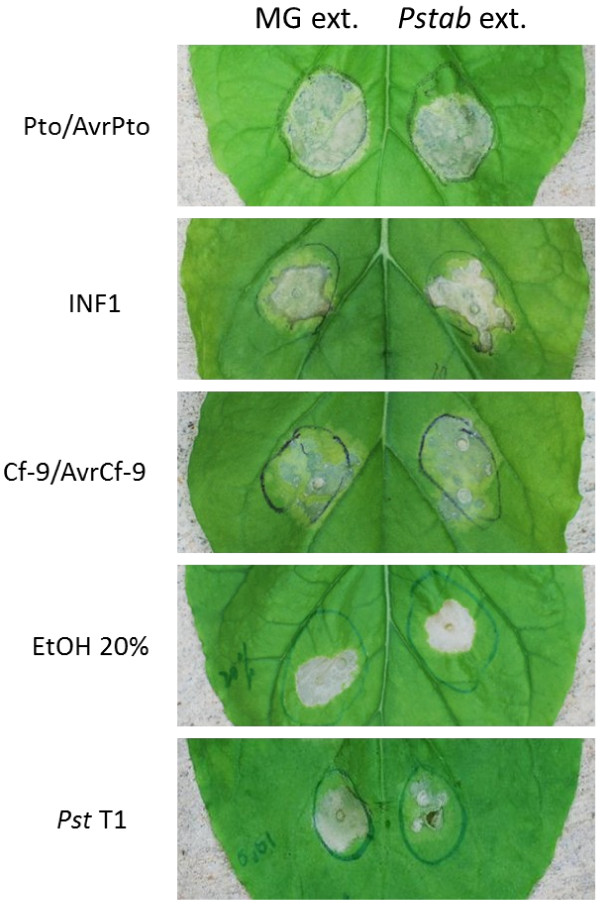
**HR cell death-induced by Avr-R interactions, PAMP, and ethanol was not altered by *Pstab *extracts in *N. benthamiana*. *N. benthamiana *leaves were infiltrated with either *Agrobacterium *carrying the binary vector that can express *INF1 *or *Cf-9 *+ *AvrCf-9 *or *Pto *+ *AvrPto*.** Photographs were taken three days after agroinfiltration for *Pto/AvrPto*, five days after inoculation for *INF1* and *Cf-9/AvrCf-9*. To determine chemical induced cell death, 20% EtOH was infiltrated and photographs were taken two days after infiltration.

To examine the stability of the metabolite, *Pstab* extracts were treated at 95°C for 5 min and infiltrated with three nonhost bacterial pathogens, *P. s.* pv. *phaseolicola*, *P. s.* pv. *maculicola* and *Pst* T1. The degree of nonhost HR cell death suppression by the boiled *Pstab* extracts did not change for all the tested nonhost pathogens (Figure 3E). However, there was cell death at the site of syringe infiltration and chlorosis symptom with the boiled *Pstab* extracts, suggesting that the virulence factor in the *Pstab* extracts may be perturbed. To investigate whether *Pstab* supernatant extracts can suppress *R-*gene- and PAMP-mediated cell death, we transiently expressed *R*-*Avr* gene combinations, *Pto*/*AvrPto*[25] and *Cf-9*/*AvrCf-9*[26], in *N. benthamiana* leaves along with *Pstab* extracts. In addition, we also expressed *INF1* (the major secreted elicitin of *Phytophthora infestans*) [27] that encodes a PAMP and causes cell death on *N. benthamiana* along with *Pstab* extracts. The development of cell death (HR) was monitored from 24 hrs after the inoculations. Interestingly, *Pstab* extracts did not suppress or reduce the intensity of cell death observed for both *R*-*Avr*-gene- and PAMP-induced HR (Figure 4). In addition, chemically induced cell death (20% EtOH) was also not affected by *Pstab* extracts, suggesting that *Pstab* extracts specifically suppress nonhost HR cell death (Figure 4).

### Metabolite profiling of the extracellular metabolites from *Pstab*

**Figure 5:**
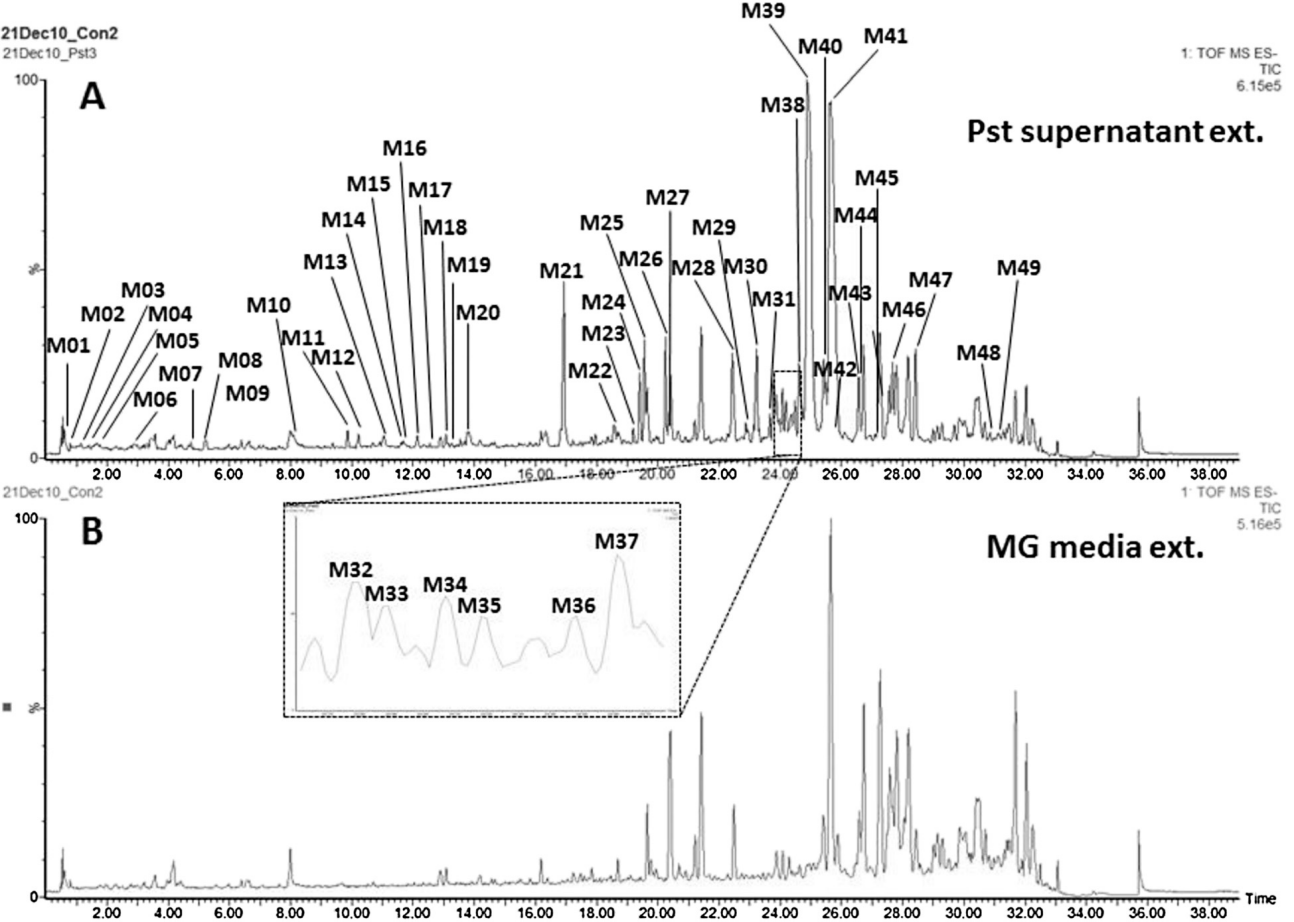
**UHPLC-qTOF-MS total ion current chromatograms (TIC) obtained in the negative electrospray ionization mode of the extracts from the *Pstab *culture supernatant (A) and the MG medium used for *Pstab *culture as the control (B).** The list of extracellular metabolites from *Pstab* is shown in Supplementary Table 1.

To further prove that *Pstab* can secrete metabolites into the growth media, we performed metabolite profiling of the *Pstab* culture media using UHPLC-qTOF-MS (Figure 5A). The extracts of the *Pstab* culture media contained secreted metabolites from *Pstab* as well as MG medium. A total of 49 extracellular metabolites from *Pstab* were detected after subtracting metabolites present in MG medium (Figure 5B). The extracellular metabolite profiling includes the retention time, specific ion (m/z) and relative intensity used to differentiate unique metabolites; however, they were not chemically annotated (Additional file 1: Table S1). In particular, M39 with the most abundant ion 1080.7322 (m/z) was quantitatively the major metabolite in the extracellular metabolites of *Pstab*.

### Altered expression of defense-related genes by *Pstab* extracts

**Figure 6:**
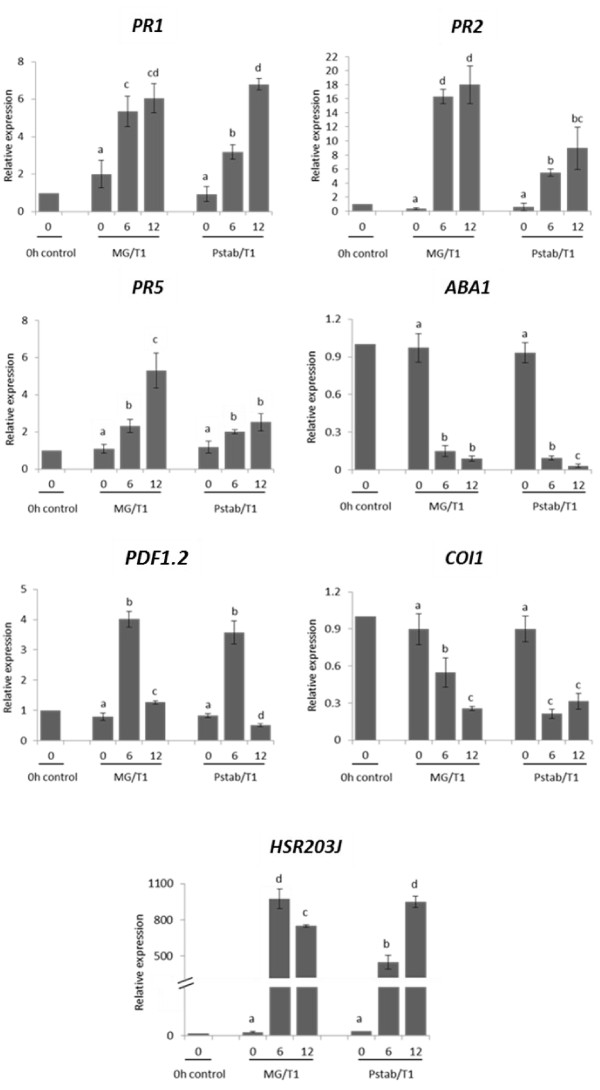
**Expression patterns of *PR1*, *PR2*, *PR5*, *PDF1.2*, *ABA1*, *COI1 *and *HSR203J *genes after inoculation with *Pst *T1 + *Pstab *extracts in *N. benthamiana*.** The expression level of *PR1*, *PR2*, *PR5*, *PDF1.2*, *ABA1*, *COI1* and *HSR203J* was determined by qRT-PCR after inoculation of *Pst* T1 with MG medium extracts or *Pstab* extracts in *N. benthamiana*. Gene expression analyses were performed using three biological replications, where each biological replicate consists of three technical replicates. Bars represent the means ± standard deviation (SD).). Same letters above bars indicate no statistically significant difference (P<0.05) among plant genotypes for a given time point using one-way ANOVA followed by LSD test analysis. The qRT-PCR data were normalized to *NbActin* transcript and shown as relative to that of target gene expressions in 0 hr *N. benthamiana* leaves without any treatment.

SA has been shown to play a role in HR cell death. We determined the gene expression of *PR1*, *PR2* and *PR5* to dissect the involvement of *Pstab* extracts in the SA-mediated defense pathway. The expression of the *PR5* gene was slightly induced in *N. benthamiana* leaves at 6 and 12 hrs after treatments of MG extracts and *Pstab* extracts. We didn’t examine for the expression of *PR1* and *PR2* after treatments of MG extracts and *Pstab* extracts. The elevation of the *PR5* expression without *Pst* T1 inoculation may be related to wounding stress by syringe infiltration. However, when treated with *Pst* T1, the expression level of *PR1*, *PR2* and *PR5* was significantly induced. Strikingly, the accumulation of *PR1*, *PR2* and *PR5* gene transcripts was significantly reduced in *N. benthamiana* leaves when the *Pstab* extracts were mixed with *Pst* T1 (Figure 6 and Additional file 2: Figure S1). We examined the expression level of *HSR203J*, the marker gene for cell death [28], after *Pst* T1 inoculation. The expression level of *HSR203J* was not different among samples treated with MG extracts or *Pstab* extracts without *Pst* T1 (Additional file 2: Figure S1). However, the expression level of *HSR203J* was significantly increased after *Pst* T1 inoculation but the induction of *HSR203J* was much lower at 6 hpi when *Pst* T1 was inoculated with *Pstab* extracts, suggesting that *Pstab* extracts delay the signaling for nonhost pathogen induced-HR cell death (Figure 6). Although, we didn’t observe any visible cell death at the site of infiltration of *Pst* T1/*Pstab* extracts, the level of *HSR203J* expression in *Pst* T1/*Pstab* extracts was similar with *Pst* T1/MG extracts at 12 hpi. These results suggest that *Pstab* extracts down-regulate SA-mediated early defense to suppress nonhost HR cell death. To determine if JA signaling is altered by *Pstab* extracts, the expression of plant defensin (*PDF1.2*), a marker for the JA signaling pathway, was examined. The expression level of *PDF1.2* was increased rapidly by 12 hrs in leaves infiltrated by MG extracts due to wound stress by syringe infiltration, while the expression level with *Pstab* extracts alone remained little changed during the experiment (Additional file 2: Figure S1), suggesting that the *Pstab* extracts suppress even the wound induced defense response. When *Pst* T1 was inoculated with MG extracts and *Pstab* extracts, the transcripts of *PDF1.2* were increased at 6 hpi and declined at 12 hpi (Figure 6). The expression pattern was not significantly different between *Pst* T1 + MG extracts and *Pst* T1 + *Pstab* extracts, suggesting that the JA-mediated defense signaling pathway against bacterial pathogen may not be directly modulated by *Pstab* extracts, in contrast to the SA-mediated pathway.

It has been known that coronatine insensitive 1 (*COI1*) gene is involved in the stomatal defense in response to bacterial pathogens [2]. Thus, we examined the gene expressions of *COI1* to determine if *Pstab* extracts target the expression of this gene. The level of expression of *COI1* gradually decreased upon infiltration of MG extracts and *Pstab* extracts alone (Additional file 2: Figure S1). This finding agrees with the results of Arabidopsis gene expressions data in the eFP Browser (http://bar.utoronto.ca/efp/cgi-bin/efpWeb.cgi) that the gene expressions are down-regulated 6 and 12 hrs after distilled water infiltration in Arabidopsis. After nonhost pathogen *Pst* T1 inoculation, *COI1* gene was significantly down-regulated by *Pstab* extracts at 6 hrs post inoculation. However, *COI1* expression pattern with *Pst* T1 inoculation appeared similar to that of the *COI1* expression pattern upon infiltration of MG extracts and *Pstab* extracts alone (Figure 6; Additional file 2: Figure S1). This finding indicates that *COI1* expression is decreased at 6 hpi by *Pstab* extracts and may interfere with the *COI1*-mediated defense pathway.

Abscisic acid (ABA) is the critical phytohormone for regulating stomatal opening and closure. *ABA1* is induced by ABA and a marker gene for the ABA signaling pathway. The expression of *ABA1* was similar to that of *COI1* where in the expression was down-regulated upon infiltration with MG extracts and *Pstab* extracts alone (Additional file 2: Figure S1). In Arabidopsis, *ABA1* expression is reduced 6 hrs after water or buffer infiltration (http://bar.utoronto.ca/efp/cgi-bin/efpWeb.cgi). *Pst* T1 infection did not alter the expression of *ABA1* compared to infiltration with MG extracts and *Pstab* extracts alone (Figure 6; Additional file 2: Figure S1). In addition, the expression pattern of *ABA1* was not significantly different among *Pst* T1 + MG extracts inoculated leaves and *Pst* T1 + *Pstab* extracts inoculated leaves.

## Discussion

Studies on the mechanisms of bacterial elicitation of early plant defense responses have unraveled important questions during plant-pathogen interactions [29-31]. It is remarkable that bacteria have evolved to deliver diverse virulence factors to defeat sophisticated plant defense systems [30,32]. Apart from the numerous proteinaceous effectors that the bacteria can deliver into plants, COR is the first discovered virulence metabolite secreted from several *P. syringae* strains and can suppress plant defense responses [2,7,10,33-35]. However, we still don’t know whether other non-COR-producing pathovars of *P. syringae* produce metabolites that have virulence function. It is still not clear how extracellular metabolites from bacterial pathogens can suppress plant defense responses.

In this study, we showed that extracellular metabolites from the non-COR producing *Pseudomonas syringae* pathogen, *Pstab*, can suppress plant defense responses including stomatal closure and nonhost HR cell death in *N. benthamiana*. Most stomata in epidermal peels of *N. benthamiana* close within 3 hrs upon inoculation with the nonhost pathogen *Pst* T1. Both *Pst* T1 and *Pstab* do not produce COR (due to lack of genes involved in biosynthesis of COR), but they can still reopen stomata in their respective host plants (Figure 2). We demonstrated that *Pstab* extracts suppress stomatal defense in *N*. *benthamiana*; however, it is unknown how *Pst* T1 suppresses stomatal defense in tomato. COR has been shown to suppress stomatal closure in Arabidopsis [2,12], and this is important for a virulent bacterium to enter the leaf apoplast. Interestingly, when a nonhost pathogen, *Pst* T1, was inoculated on *N. benthamiana* with *Pstab* extracts, the stomatal closure induced by *Pst* T1 was suppressed (Figure 2). This finding suggests that the non-COR-producing strain *Pstab* may produce other uncharacterized extracellular metabolites that may have a similar function as COR in opening stomata.

We also found that the nonhost HR cell death induced by *Pst* T1 in *N. benthamiana* was also suppressed when inoculated with *Pstab* extracts (Figure 3). It has been known that *Pstab* produces tabtoxin that induces chlorosis and is associated with the symptoms of wildfire disease on tobacco and halo blight of oats [36]. Tabtoxin is a dipeptide which is not biologically active and needs to be hydrolyzed by peptidase for an active toxic component, tabtoxinine-β-Lactam (TβL). TβL is released by zinc-dependent aminopeptidases in the periplasm of the bacterium or by enzymes in the plant cell, and inhibits the enzyme glutamine synthetase that results in chlorosis [37,38]. Raaijmakers et al. [39] described that TβL is proved to be difficult to synthesize due to the toxin’s instability. Tabtoxin is a relatively unstable molecule *in vivo* and during the procedures of toxin purification [40,41]. It has been demonstrated that the biological activity of a tabtoxin solution decreases with a half-life of approximately one day at room temperature [41]. In addition, chlorosis is the most characteristic symptom of tabtoxin in tobacco leaves [42]. We, therefore, hypothesized that *Pstab* extracts does not contain tabtoxin. To prove that metabolites but not active proteins play a role in stomata opening and HR suppression, the *Pstab* culture supernatant was extracted using an organic solvent, ethyl acetate with 1% formic acid. The process of ethyl acetate extraction removes peptides from *Pstab*. We also examined whether the *Pstab* extracts have any toxic activity in *N. benthamiana* leaves. The inoculated leaves did not produce any visible symptoms contrary to tabtoxin that produces yellowing (Figure 3A) [43].

*P. syringae* pathovars produce several non-effector type virulence factors such as tabtoxin, phaseolotoxin, mangotoxin, and COR [7]. All these phytotoxins except COR are made of small peptide molecules. COR is a non-proteinaceous phytotoxin that is involved in stomata opening and suppressing SA-dependent host defense responses [35]. COR was isolated from *P. syringae* pv. *tomato* using HPLC fractionation [7]. However, we used a metabolomics approach to isolate bioactive metabolite(s) from *Pstab* extracts using UHPLC-qTOF-MS for metabolite profiling. The metabolite profiling determined the total number of metabolites secreted from *Pstab* in the bacterial culture. This metabolite profiling technique has been effectively used to isolate metabolites differentially expressed in different bacterial strains [44]. Since there were not a large number of metabolites (Figure 5), we can further examine bioactive metabolite(s) which can suppress stomatal closure and nonhost HR cell death using HPLC fractionation. The identification of the *Pstab* extracellular metabolite(s) that suppresses early plant defense responses in the future will advance our understanding of bacterial pathogen-plant infections.

Salicylic acid (SA) is an important plant hormone for regulating defense responses like stomatal closure and HR [3,45]. The bacterial growth of both nonhost pathogens, *Psgly* and *Pst* T1, was significantly higher in *N. benthamiana* when they were co-inoculated with *Pstab* extracts (Figure 3). This could be due to suppression of SA-mediated early defense signaling (Figure 6) by *Pstab* extracts or due to the suppression of HR. The level of nonhost bacterial population gradually decreased three days after inoculation, and no disease symptoms appeared on the inoculated leaves (Figure 3A and 3B) suggesting that *Pstab* extracts suppress only early defense responses but do not function as pathogenicity factors.

We determined whether *Pstab* extracts also modulate the jasmonic acid (JA)-mediated defense pathway by examining the level of *PDF1.2* and *COI1* gene expression. The bacterial virulence factor COR stimulates the JA pathway, thereby suppressing the SA pathway in Arabidopsis and tomato [11,35]. It has been shown that COR targets *COI1* (F-box subunit of the SCFCOI1 ubiquitin ligase)/*JAI1* (Jasmonic Acid Insensitive 1)-dependent pathways to promote suppression of stomatal-based immunity [3,46]. JAZ proteins are repressors of JA signaling, and few JAZ proteins (JAZ1, JAZ3, and JAZ9) have been shown to interact with *COI1*[47]. It has also been shown that *COI1* plays a role in plant cell death during plant-microbe interaction. When *COI1* was silenced in *N. benthamiana*, cell death-induced by potato virus X (*PVX*) was accelerated [48]. Devadas et al. [49] reported that a *hrl1* (hypersensitive response-like lesions 1) *coi1* double mutant exacerbated cell death lesions, suggesting that *COI1* is necessary for cell death limitation.

Interestingly, in our study, we observed that nonhost HR cell death was suppressed by *Pstab* extracts, but not for any of the specific R-AVR or PAMP triggered cell death. It is possible that the unknown metabolite in *Pstab* extracts may specifically inhibits the defense mechanism involved in nonhost resistance. Further studies should be performed to isolate the metabolite and identity its function for the suppression of nonhost HR cell death in response to bacterial pathogens.

## Conclusions

It is demonstrated here that another *Pseudomonas* pathogen, *Pstab* that does not produce COR, can still actively suppress stomatal defense and nonhost HR cell death in *N. benthamiana*. This finding clearly suggests that *Pseudomonas syringae* strains can produce metabolite(s) other than COR to suppress plant defense responses. Isolation and characterization of the *Pstab* extracellular metabolite(s) will facilitate a better understanding of strategies used by bacterial pathogens to cause disease in host plants.

## Methods

### *N. benthamiana* growth

*N. benthamiana* was sowed on soilless potting mix BM7 (Berger Co., Quebec, Canada) and grown in a growth room at 22 to 25°C for two weeks under a 12 hr photoperiod with light intensity ranging from 300 to 400 μmol m^-2^ s^-1^. Two-week-old seedlings were transplanted to 10 cm diameter round pots containing potting soil (BM7) (Berger Co., Quebec, Canada), with one plant per pot, and grown in the greenhouse at 23°C with 16 hrs of extended day-light and supplemental lighting at 100 μmol m^-2^ s^-1^. Plants were regularly fertilized (20-10-20). Three- to four-week-old *N. benthamiana* plants were used for the experiments.

Seeds of tomato (*Solanum lycopersicum* cv. Glamour) were obtained from Stokes Seeds Inc (Buffalo, NY, USA). Plants were grown in Scott-200® mix (The Scotts Co., Marysville, Ohio, U.S.A.) and maintained in growth chambers (24°C, 40-70% RH, 12 h photoperiod, photon flux density of 150–200 μmol m^-2^ sec^-1^). Three-week-old seedlings were transplanted to 10 cm diameter round pots and maintained the same as the *N. benthamiana* plants described above.

### Metabolite extracts from the *Pstab* culture supernatant

To extract metabolites secreted from *Pstab*, we followed the extraction method used for COR with minor modifications [50]. The bacterial strain *Pstab* was grown on a KB plate containing appropriate antibiotics. A single colony of *Pstab* was incubated in 10 ml of Manitol-Glutamate (MG) medium (manitol, 10 g; L-glutamic acid, 2 g; KH_2_PO_4_, 0.5 g; NaCl, 0.2 g; MgSO4, 0.2 g with pH7/liter) at 28°C with 250 rpm for 48 hr. The 2.5 ml of *Pstab* culture was added to 47.5 ml fresh MG medium and cultured for six days at 18°C with 220 rpm in a rotary shaker. The *Pstab* cultures (150 ml) were centrifuged for 30 min at 3,700×*g* at 4°C, and the supernatant was transferred to a sterile glass tube. The *Pstab* cell pellets were used for analysis of intracellular metabolites and the supernatant for analysis of extracellular metabolites. For extraction of intracellular metabolites, the *Pstab* cell pellets were placed in a glass vial with 5 ml ethyl acetate containing 1% (v/v) formic acid and then sonicated for 20 min. The ethyl acetate fraction was collected through centrifugation (3,700×*g*, 30 min) and completely dried, using N_2_ gas. For extraction of extracellular metabolites, the *Pstab* supernatant was extracted with ethyl acetate containing 1% formic acid. The ratio of the culture supernatant to the acidified ethyl acetate was 3:5. The ethyl acetate fraction was collected and completely dried, using N_2_ gas. The MG medium without *Pstab* was extracted as a control, following the above procedures. The dried extracts were resuspended in 600 μl of 16.7% methanol in H_2_O. For stomatal and nonhost HR cell death bioassays, 6 μl of extracts were used for every milliliter of inoculation buffer (10 mM MES, pH 6.5).

### Assays for suppression of stomatal closure by *Pstab* extracts

*Pst* T1 was grown in KB medium overnight at 28°C. The bacterial culture was centrifuged at 3,500 rpm for 10 min and resuspended in nanopure water at a concentration of 1×10^7^ CFU/ml. For assay of the bacterial migration through stomata, detached *N. benthamiana* leaves were floated on a bacterial suspension of *Pst* T1 containing MG media extract or the *Pstab* extracts for 1 to 4 hrs. After the incubation, leaf disks were collected and a number of bacterial cells in the apoplast were examined. This experiment was repeated three times under the same conditions. For stomatal closure assay, we followed the epidermal peel assay as described [2,14]. To assure that most stomata are open before beginning experiments, plants were kept under light for 3 hrs, and the epidermis of *N. benthamiana* leaves was peeled off and immediately floated on stomata opening buffer (10 mM MES-KOH, 30 mM KCl, pH6.3) for 3 hrs. At various time points, the epidermal peels were treated with pathogens, chemicals and bacterial secreted metabolites. An average of 100 random stomatal apertures was measured each treatment, and three samples were collected from each experiment with two replications.

### Assays for suppression of nonhost HR cell death by *Pstab* extracts

Five *P. syringae* species, one host (*Pstab*) and four nonhost pathogens [*Pst* T1, *P. s.* pv. *glycinea* (*Psgly*), *P. s.* pv. *phaseolicola* (*Psp*) and *P. s.* pv. *maculicola* (*Psm*)] were used for this experiment. The bacterial pathogens were cultured in KB medium with appropriate antibiotics overnight at 28°C on a rotary shaker (250 rpm). Bacteria were collected by centrifugation (3500 rpm/10 min) and resuspended in MES buffer (MES 10 mM, pH 6.5). The bacterial suspension was syringe-infiltrated to fully expanded *N. benthamiana* leaves for determining bacterial growth (for *Psgly*) and nonhost HR cell death (for *Pst* T1). To determine whether the bacterial growth is promoted by *Pstab* extracts, the nonhost pathogen *Psgly* that does not induce HR cell death was inoculated with either MG medium or the *Pstab* extracts. The bacterial population in the apoplast was examined for 0, 1, 3 and 5 days. For nonhost HR cell death assay, each metabolite sample was infiltrated with *Pst* T1 using a needle-less syringe on six-week-old *N. benthamiana* leaves. We selected fully extended upper leaves for the inoculation. In all experiments, the extracts from *Pstab* supernatant and MG medium were included as positive and negative controls. To eliminate any contaminating proteins in *Pstab* extracts, the extracts were treated at 95°C for 5 min. Two additional nonhost bacterial pathogens, *Psp* and *Psm*, including *Pst* T1 were inoculated along with the boiled *Pstab* extracts in *N. benthamiana*. Symptoms of nonhost HR cell death were determined from 12 hrs to 72 hrs after inoculation.

To determine the specificity of *Pstab* extracts suppressing nonhost HR cell death, we used several *Avr*-*R* gene combinations (35S:*AvrPto*-35S:*Pto* and 35S:*Avr9*-35S:*Cf9*) [51,52]. *Agrobacterium* containing the respective constructs were cultured in 5 mL LB medium containing respective antibiotics for 24 hr at 28°C. The bacterial culture was centrifuged at 1,500 g for 10 min and resuspended in 5 mL of infiltration buffer (10 mM morpholinoethane sulfonic acid (MES) and 200 mM acetosyringone). Then, the culture was incubated at room temperature for three to five hrs. After incubation, bacterial cells were harvested by centrifugation and diluted to 5×10^5^ CFU/ml (*inf1*, *Pto*, and *AvrPto*) and 2×10^7^ CFU/ml (*Cf9* and *AvrCf9*) for infiltration. *Pto* and *AvrPto*, and *Cf9* and *AvrCf9* constructs were mixed to 1:1 ratio before infiltration to *N. benthamiana* leaves.

### Metabolite profiling of the *Pstab* supernatant extracts

The dried *Pstab* extracts were resuspended in 150 μl of 80% methanol in H_2_O and analyzed using a UHPLC-ESI(-)-qTOF-MS instrument (Waters Premier qTOF) with a reverse-phase column (ACQUITY UPLC™ BEH C18 1.7 μm, 2.1 mm × 150 mm), which was maintained at 60°C, and components were eluted using a multi-step gradient from 95 to 30% A (eluent A, 0.1% aqueous acetic acid) over 30 min, 30 to 5% over 3 min and 5 to 95% A over 3 min at a flow rate 0.56 mL/min. The complementary eluent B was acetonitrile. TOF-MS spectra were acquired under the following conditions: spectral acquisition rate: 3.13 per second; detector voltage: 2600 V; threshold: 2037; ESI: -4500 V; desolvation temperature: 300°C; nebulizer pressure: 350 kPa; interface: 100°C. Mass measurement accuracy was within 20 ppm. The MS system was calibrated using sodium formate, and raffinose was used as the lock mass for internal calibration. Data obtained from metabolite analyses were processed using MarkerLynx 4.1 (Waters) for accurate data mass extraction. Relative abundance was normalized by dividing each peak area by the value of the internal standard peak area.

### Determination of defense signaling modulated by *Pstab* extracts

To examine the expression patterns of genes involved in SA-, JA- and ABA-related plant signal transduction pathways, *N. benthamiana* leaf samples were collected at various time points after treatments. RNA was isolated from leaves at 0, 6 and 12 hrs after inoculation with *Pst* T1 + *Pstab* extracts (*Pstab* ext./*Pst* T1) or with *Pst* T1 + MG medium extracts as control (MG ext./*Pst* T1). All RNA samples were extracted using Qiagen RNeasy Mini Kit (Qiagen, Valencia Calif.), and cDNA was synthesized using Superscript III reverse transcriptase (Invitrogen, Grand Island, N.Y.). Quantification and purity of RNA and cDNA were determined using NanoDrop (Thermo Scientific, Wilmington, Del.). Expressions of *PR1*, *PR2*, *PR5, PDF1.2, ABA1,* and *COI1*, representative genes for each ABA, SA and JA signal pathway, and *HSR203J* for HR cell death, were determined by quantitative real-time PCR. At least three biological replicates of each sample and three technical replicates of each biological replicate were analyzed for real-time PCR analysis. The amount of transcripts for each gene in different RNA samples was normalized with the transcripts of the internal control gene *NbActin* to ensure an equal amount of cDNA was used for individual reactions.

## Competing interests

The authors declare that they don’t have any competing interests.

## Authors’ contributions

SL performed major parts of the experiments; DY prepared *Pstab* extracts and carried out mass-spectrometry analysis for *Pstab* extracts; SRU carried out confocal images of GFP*uv*-expressing bacteria in stomata and apoplast space; LWS designed bacterial metabolomics work and edited this manuscript; KSM participated in designing and coordinating the project. SL and KM wrote the manuscript. All authors read and approved the final manuscript.

## Supplementary Material

Additional file 1: Table S1 List of extracellular metabolites secreted from *P. syringae* pv. *tabaci* using UHPLC-qTOF-MS. Click here for file

Additional file 2: Figure S1 Expression patterns of *PR5*, *PDF1.2*, *ABA1*, *HSR203J* and *COI1* genes after treating with MG or *Pstab* extracts in *N. benthamiana*. The expression level of *PR5*, *PDF1.2*, *ABA1*, *HSR203J* and *COI1* as determined by qRT-PCR after treatment of *Pstab* extracts. Gene expression analyses were performed using three biological replications. Bars represent the means ± standard deviation (SD). Same letters above bars indicate no statistically significant difference (P<0.05) among plant genotypes for a given time point using one-way ANOVA followed by LSD test analysis. The qRT-PCR data were normalized to *NbActin* transcript and shown as relative to that of target gene expressions in 0 hr *N. benthamiana* leaves without any treatment. Click here for file
