# Supplementary material for: Suppression of plant defense responses by extracellular metabolites from Pseudomonas syringae pv. tabaci in Nicotiana benthamiana
Source: BMC Plant Biol. 2013 Apr 18;13:65. doi: 10.1186/1471-2229-13-65 (PMC3648423; doi:10.1186/1471-2229-13-65)
Supplement: Additional file 1: Table S1 — List of extracellular metabolites secreted from P. syringae pv. tabaci using UHPLC-qTOF-MS. [file 1471-2229-13-65-S1.doc]

**Additional material**

**Additional file 1: Table S1.** List of extracellular metabolites secreted from *P. syringae* pv. *tabaci* using UPLC-qTOF-MS.

|  | | | |
| --- | --- | --- | --- |
| ID | Ret. Time  (min) | Specific ion  (m/z) | Relative abundance *^a^*  (mean ± S.D.) |
| M01 | 0.5956 | 195.0489 | 0.4±0.4 |
| M02 | 0.8025 | 173.0091 | 0.0±0.0 |
| M03 | 1.1410 | 274.0930 | 0.4±0.1 |
| M04 | 1.3853 | 242.0610 | 0.1±0.0 |
| M05 | 1.6650 | 149.0268 | 0.2±0.0 |
| M06 | 2.8464 | 433.1774 | 0.1±0.1 |
| M07 | 4.7387 | 213.1224 | 0.8±0.1 |
| M08 | 5.2107 | 313.1640 | 0.2±0.2 |
| M09 | 5.2111 | 475.2239 | 0.2±0.2 |
| M10 | 8.0827 | 341.1939 | 0.5±0.3 |
| M11 | 9.8489 | 285.9983 | 0.4±0.5 |
| M12 | 10.1967 | 285.9985 | 0.3±0.4 |
| M13 | 11.0290 | 369.2255 | 0.5±0.5 |
| M14 | 11.6025 | 513.2695 | 0.1±0.0 |
| M15 | 11.6572 | 393.2250 | 0.1±0.2 |
| M16 | 12.1076 | 513.2670 | 0.7±0.1 |
| M17 | 12.5286 | 797.5126 | 0.5±0.3 |
| M18 | 13.0369 | 294.1191 | 0.2±0.0 |
| M19 | 13.3586 | 811.5298 | 0.0±0.0 |
| M20 | 13.7978 | 397.2558 | 1.6±0.9 |
| M21 | 16.9205 | 300.2179 | 8.0±3.5 |
| M22 | 18.5728 | 477.3209 | 1.8±1.8 |
| M23 | 19.1718 | 755.4923 | 1.7±0.7 |
| M24 | 19.3988 | 755.4939 | 9.3±3.4 |
| M25 | 19.5483 | 413.3024 | 8.3±5.0 |
| M26 | 20.2478 | 654.4451 | 12.0±4.1 |
| M27 | 20.3484 | 625.3951 | 2.4±0.3 |
| M28 | 22.4170 | 1052.7020 | 10.8±1.5 |
| M29 | 22.8501 | 967.6469 | 1.6±0.3 |
| M30 | 23.2166 | 1066.7189 | 11.2±2.8 |
| M31 | 23.6486 | 1082.7130 | 2.3±0.8 |
| M32 | 23.7981 | 1052.7023 | 977.7±321.2 |
| M33 | 23.8030 | 1098.6910 | 3346.4±924.9 |
| M34 | 24.0222 | 1066.7180 | 3516.5±987.8 |
| M35 | 24.1658 | 1098.6885 | 1400.9±598.7 |
| M36 | 24.4832 | 1112.7070 | 6.1±3.7 |
| M37 | 24.6070 | 1066.7189 | 2952.4±212.7 |
| M38 | 24.6873 | 1080.7346 | 631.6±191.2 |
| M39 | 24.9161 | 1080.7322 | 62350.4±3312.9 |
| M40 | 25.4065 | 1080.7339 | 2924.5±177.7 |
| M41 | 25.6406 | 1094.7478 | 129.0±37.2 |
| M42 | 25.8380 | 1108.7308 | 2.6±0.9 |
| M43 | 26.5595 | 1122.7412 | 1.9±0.3 |
| M44 | 26.5675 | 1106.7501 | 3.6±0.1 |
| M45 | 27.0767 | 565.4438 | 0.5±0.5 |
| M46 | 27.6578 | 1108.7665 | 10.3±1.5 |
| M47 | 28.4019 | 1122.7822 | 9.5±1.9 |
| M48 | 30.9013 | 851.5392 | Tr*^b^* |
| M49 | 31.1741 | 187.1323 | 0.3±0.1 |
| *^a^* Relative abundance was normalized to the area of the internal standard.  *^b^* Trace amount | | | |
