# Supplementary material for: Suppression of plant defense responses by extracellular metabolites from Pseudomonas syringae pv. tabaci in Nicotiana benthamiana
Source: BMC Plant Biol. 2013 Apr 18;13:65. doi: 10.1186/1471-2229-13-65 (PMC3648423; doi:10.1186/1471-2229-13-65)
Supplement: Additional file 2: Figure S1 — Expression patterns of PR5, PDF1.2, ABA1, HSR203J and COI1 genes after treating with MG or Pstab extracts in N. benthamiana. The expression level of PR5, PDF1.2, ABA1, HSR203J and COI1 as determined by qRT-PCR after treatment of Pstab extracts. Gene expression analyses were performed using three biological replications. Bars represent the means ± standard deviation (SD). Same letters above bars indicate no statistically significant difference (P<0.05) among plant genotypes for a given time point using one-way ANOVA followed by LSD test analysis. The qRT-PCR data were normalized to NbActin transcript and shown as relative to that of target gene expressions in 0 hr N. benthamiana leaves without any treatment. [file 1471-2229-13-65-S2.pptx]

## Slide 1
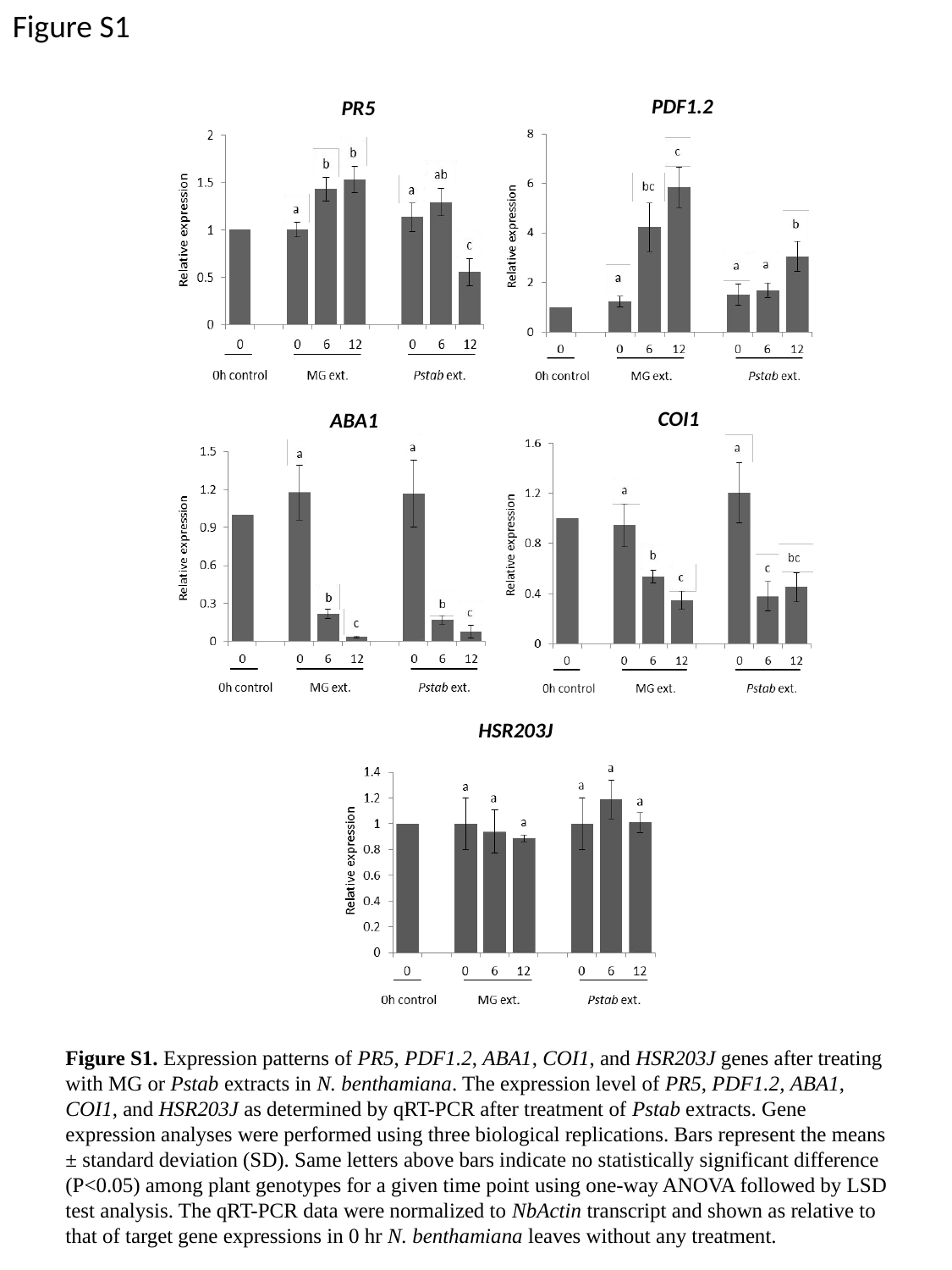

Figure S1
PR5
PDF1.2
COI1
ABA1
HSR203J
Figure S1. Expression patterns of PR5, PDF1.2, ABA1, COI1, and HSR203J genes after treating with MG or Pstab extracts in N. benthamiana. The expression level of PR5, PDF1.2, ABA1, COI1, and HSR203J as determined by qRT-PCR after treatment of Pstab extracts. Gene expression analyses were performed using three biological replications. Bars represent the means ± standard deviation (SD). Same letters above bars indicate no statistically significant difference (P<0.05) among plant genotypes for a given time point using one-way ANOVA followed by LSD test analysis. The qRT-PCR data were normalized to NbActin transcript and shown as relative to that of target gene expressions in 0 hr N. benthamiana leaves without any treatment.
